# Parallel chemical switches underlying pollinator isolation in Asian *Mitella*

**DOI:** 10.1111/jeb.12591

**Published:** 2015-02-20

**Authors:** T Okamoto, Y Okuyama, R Goto, M Tokoro, M Kato

**Affiliations:** * Graduate School of Human and Environmental Studies, Kyoto UniversitySakyo, Kyoto, Japan; † Forestry and Forest Products Research InstituteTsukuba, Ibaraki, Japan; ‡ Tsukuba Botanical Garden, National Museum of Nature and ScienceTsukuba, Ibaraki, Japan

**Keywords:** floral scent, independent contrast, phylogenetics, pollination, reproductive isolation, speciation

## Abstract

Floral scents are among the key signals used by pollinators to navigate to specific flowers. Thus, evolutionary changes in scents should have strong impacts on plant diversification, although scent-mediated plant speciation through pollinator shifts has rarely been demonstrated, despite being likely. To examine whether and how scent-mediated plant speciation may have occurred, we investigated the *Asimitellaria* plant lineage using multidisciplinary approaches including pollinator observations, chemical analyses of the floral scents, electroantennographic analyses and behavioural bioassays with the pollinators. We also performed phylogenetically independent contrast analyses of the pollinator/floral scent associations. First, we confirmed that the pairs of the sympatric, cross-fertile *Asimitellaria* species in three study sites consistently attract different pollinators, namely long-tongued and short-tongued fungus gnats. We also found that a stereoisomeric set of floral volatiles, the lilac aldehydes, could be responsible for the pollinator specificity. This is because the compounds consistently elicited responses in the antennae of the long-tongued fungus gnats and had contrasting effects on the two pollinators, that is triggering the nectaring behaviour of long-tongued fungus gnats while repelling short-tongued fungus gnats in a laboratory experiment. Moreover, we discovered that volatile composition repeatedly switched in *Asimitellaria* between species adapted to long-tongued and short-tongued fungus gnats. Collectively, our results support the idea that recurrent scent-mediated speciation has taken place in the *Asimitellaria*–fungus gnat system.

## Introduction

A tremendous diversity of animal-pollinated plants, perhaps exceeding 300 000 species (Ollerton *et al*., 2011), provides unparalleled opportunities to examine the adaptive changes involved in speciation (Rieseberg & Willis, 2007). Specifically, it is hypothesized that changes in floral traits perceived by pollinators should cause divergence in pollinator assemblages and ultimately lead to the formation of reproductive barriers between previously interbreeding plant populations (Johnson, 2010). Although this ‘speciation through pollinator shift’ model is widely recognized, whether pollinator shifts have played primary roles for establishing reproductive barriers between recently diverged populations is still under debate (Johnson, 2010). Moreover, because pollinator isolation often involves a coordinated set of floral traits involving colour, reward and scent, explaining how changes in individual traits contributed to the formation of isolation barriers is difficult (Bradshaw & Schemske, 2003).

Among the floral traits, the roles of floral scent evolution in the initial stages of plant speciation have only recently attracted attention (Schiestl & Ayasse, 2002; Waelti *et al*., 2008; Anderson *et al*., 2010; Shuttleworth & Johnson, 2010). The great variation in floral volatiles, even within closely related plant species, suggests that scent has a major role in determining the range of pollinators on which plants depend (Raguso & Pichersky, 1995; Xu *et al*., 2012). Nevertheless, its critical role in the formation of isolating barriers is only demonstrated in a nonrewarding, sexually deceptive plant group (Peakall *et al*., 2010; Dell'Olivo *et al*., 2011; Xu *et al*., 2011; Whitehead & Peakall, 2014). In this study, we report a novel case of recurrent chemical changes paralleled with pollinator switching in the *Asimitellaria*–fungus gnat pollination system. The system will provide further insights into the mechanisms of possible scent-based floral isolation in a rewarding system.

**Fig 1:**
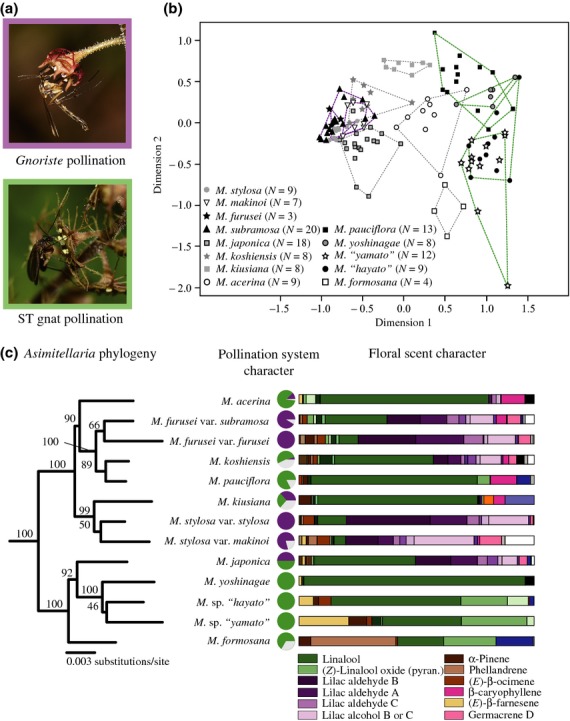
Divergent pollination systems and associated floral scent profiles in *Asimitellaria*. (a) Pollination by *Gnoriste mikado* (purple frame) and short-tongued (ST) gnats (green frame). (b) Nonmetric multidimensional (NMDS) ordination plot of 128 floral scent samples. Different symbols indicate different plant species. Samples are bounded by either purple, green or grey lines indicating species pollinated specifically by *G. mikado,* short-tongued gnats or both, respectively. (c) Pollination system characters and floral scent characters of 13 *Asimitellaria* species; phylogeny of the 13 species is also presented. Numbers above branches are bootstrap values. The characteristics of the pollination system are presented in pie charts; purple, green and white slices indicate the relative frequency of pollination by *G. mikado*, short-tongued gnats and other dipterans, respectively. Floral scent characters are presented as bar charts using data in Table S1; differently coloured areas indicate different compounds.

*Mitella* sect. *Asimitellaria* (Saxifragaceae; *Asimitellaria* throughout the present paper) is a monophyletic group of perennials endemic to Japan and Taiwan. *Asimitellaria* species flower in spring and almost exclusively depend on a nectar-rewarding system with two types of pollinators, one of which is a long-tongued fungus gnat species (*Gnoriste mikado* Okada) and the other comprises short-tongued fungus gnats (a group of species consisting mostly of closely related *Boletina* spp. and *Coelosia* sp. [Mycetophilidae]) (Fig.1a). The species of *Asimitellaria* vary by the extent to which they depend on these alternative pollinators. Some are pollinated specifically by *G. mikado*, some by short-tongued gnats and others by both (Okuyama *et al*., 2004, 2008; Okuyama, 2012).

We hypothesized that this variation in the pollination system may have arisen from differences in floral scent. Accordingly, we asked the following questions in this study: Is pollinator specificity strict even under sympatric occurrences of the *Asimitellaria* species, and if yes, does any floral scent chemical basis for pollinator specificity, and any chemical changes parallel with pollinator shifts, exist? Finally, we briefly discuss the role of floral scent changes in plant speciation and explore the possibility that pollinator differences function as the principal isolating barrier between the *Asimitellaria* species.

## Materials and methods

### Pollinator data

For convenience, we regard taxonomic varieties and undescribed species as distinct species (or evolutionary unit) throughout this study, as each of them is genetically differentiated from each other (Okuyama & Kato, 2009). Specifically, ribosomal external- and internal-transcribed spacer sequence divergences exceeding those commonly found within species have been found in this group, confirming it consists of a complex of genetically distinct but morphologically cryptic species (Okuyama & Kato, 2009).

Data on the pollinators for all 13 *Asimitellaria* ‘species’ (Fig.1) except one (*Mitella yoshinagae* H. Hara) have been reported in previous field studies (Okuyama *et al*., 2008; Okuyama, 2012). Pollinators were categorized into three functional groups as follows: long-tongued *G. mikado*, short-tongued fungus gnats and other dipterans. The pollinators of *M. yoshinagae* were determined following a 4-h direct observation of 16 plants at Sade, Mima, Tokushima Prefecture, Japan (site 1) on 9 and 12 April 2007. We scored the relative proportions (%) of the respective pollinator categories recorded on flowers of each species as the species-specific pollination character. Per cent was used because it was the standardized value only available in the present comparative analysis.

Furthermore, to confirm pollinator specificity in sympatric populations of two *Asimitellaria* species, we monitored flower visits by direct observation or by time-lapse photography. Many of the *Asimitellaria* species have very limited distribution ranges, and there are only limited pairs of sympatric species. Among them, we chose three pairs, all of which are interfertile if crossed artificially (Okuyama & Kato, 2009). Specifically, we include the pair of closely related species, *Mitella pauciflora* and *Mitella furusei* var. *subramosa,* to clarify whether the pollinator specificity is strict even between the closely related species. This is also the most commonly observed, sympatric *Asimitellaria* species pair in wild because the two species have widely overlapping distributions in western Japan. During direct observations, two observers simultaneously recorded visitors to the flowers of two sympatric *Asimitellaria* species, each growing in clusters < 15 m apart. Observations of *M. yoshinagae* and *Mitella stylosa* H. Boissieu var. *makinoi* (H. Hara) Wakab. were conducted over 2 h on 12 April 2007 at site 1 and of *Mitella ‘yamatoensis’* and *M. furusei* Ohwi var. *subramosa* Wakab. over 6 h on 26 April and 1 and 2 May 2006 at the Akame 48 Waterfalls, Nabari, Mie Prefecture, Japan (site 2); on all occasions, we maintained a distance > 1 m from the plants. Each landing of a flower-visiting insect on an inflorescence was recorded as a single visit, and the same individual insect was not counted again until it had left the inflorescence. Time-lapse photography over 2-min intervals (Okuyama, 2012) was conducted through the days and nights of 8, 9, 13–16, 19–22 and 26 April 2010 at Kibune, Kyoto, Kyoto Prefecture, Japan (site 3), for a total of 132 h. We used two digital cameras (Optio W80; Pentax, Tokyo, Japan) each set > 20 cm away from individual plants of *M. pauciflora* Rosend. or *M. furusei* var. *subramosa*; the two cameras photographing an individual plant were < 5 m from each other and focused on 1–4 inflorescences. Each time an insect individual was photographed gathering nectar on a flower, we recorded it as a single visit; observations of the same insect on the same inflorescence (judged from those captured in the subsequent images) were not incorporated into the data set. However, even the case where the same insect might have visited different inflorescences of the same plant was counted as an individual visit. Identification of the insects was straightforward because the flower-visiting fungus gnats are different from the other dipterans in their medium-sized (5–6 mm), characteristic body morphology, and *G. mikado* is unique among the fungus gnats in its larger body size (7–8 mm), brownish wings and a long proboscis.

Every pair of *Asimitellaria* species in these study sites is distinct in multiple traits both in vegetative and in floral parts (Wakabayashi, 2001). Furthermore, we have previously created many combinations of interspecific hybrids including those between the species studied in this study (Okuyama & Kato, 2009). We knew from this work that hybridization generates recognizable and morphologically intermediate forms between the parental species. Based on this knowledge, we searched for potential plant hybrids at the time of the study visits.

### Collection and analysis of floral volatiles

We examined the floral scent traits of 13 *Asimitellaria* species by collecting floral volatiles from 128 potted plants (3–20 individuals per species) using a headspace adsorption technique (Raguso & Pellmyr, 1998). The *Asimitellaria* plants used for scent collection were all collected in the wild, transplanted to a pot containing well-fertilized soil, and cultivated in a garden at Kyoto University for at least 5 months until the day of floral scent collection. Floral scents were collected directly from inflorescences of potted plants in a clean room set at 20 °C and held under artificial illumination. For individual plants, only the inflorescences (without being cut off from the plants) were covered with a glass bottle to obtain the floral headspace. A glass tube filled with 60 mg of Tenax TA (SIS, Ringoes, NJ, USA) absorbent resin (preconditioned under nitrogen at 300 °C for 2–3 h) was connected through a Tygon® (Saint-Gobain, Aurora, OH, USA) tube to the glass bottle. Air containing floral volatiles was pumped with a mini air pump (MP-2N; SIBATA, Tokyo, Japan) for 2 h between 16:00 and 20:00 at an airflow rate of 200 mL min^−1^ through the tube. As a control, we simultaneously collected the volatiles from ambient air. The glass tube containing captured floral volatiles was sealed with glass beads, wrapped with aluminium foil and stored at –30 °C until the contents were analysed.

Subsequently, the scent samples were subjected to gas chromatography/mass spectrometry (GC/MS) (QP2010; Shimadzu, Kyoto, Japan). Volatiles were eluted from the adsorbent with 2 mL of diethyl ether; 1 μL of *n*-hexadecane (1 mg mL^−1^) was added as an internal standard. The eluate was concentrated with a N_2_ flow to 10 μL and topped up with 10 μL of hexane. An aliquot (1 μL) of each sample was used for GC/MS. For GC, we used an Rtx-5SilMS capillary column (30 m × 0.25 mm; film thickness, 250 μm; Restek, Bellefonte, PA, USA). Helium was used as the carrier gas at a velocity of 48.1 cm s^−1^, and the injector temperature was 250 °C. The injector was operated in the splitless mode for 1 min. Electron ionization mass spectra were obtained at an ionization voltage of 70 eV and a source temperature of 200 °C. The oven temperature was programmed to the following sequence: 40 °C for 5 min, followed by an increase of 5 °C min^−1^ to 280 °C, at which the oven was held for 5 min. For preliminary identification of the compounds, we compared the fragments to those contained in the National Institute of Standards and Technology (Gaithersburg, MD, USA) NIST 05 libraries. We isolated only volatiles emitted from flowers by comparing volatiles detected from flower samples with those from control samples. The retention indices for all compounds were calculated with n-alkane (C9–C20) standards and were compared with those reported in the NIST Chemistry WebBook (Linstrom & Mallard, 2012) and The Pherobase (El-Sayed, 2008). Whenever possible, we further verified identification of a subset of the compounds using the retention indices and mass spectrum fragments of authentic compounds. The percentage composition of each volatile compound was obtained by calculating its peak area as a percentage of the total peak area on the gas chromatograms, and the quantity of each volatile compound was calculated by comparing the GC data with the internal standards. Authentic compounds were purchased from Wako Pure Chemical (Osaka, Japan), Nakarai Tesque (Kyoto, Japan), TCI (Tokyo, Japan) and Sigma-Aldrich (Tokyo, Japan), unless stated otherwise.

Finally, among the compounds detected, we regarded 6-methyl-5-hepten-2-one, geranyl acetone, nonanal and decanal as the artificial contamination and excluded from the data set, as it has been frequently reported that they are generated via nonbiological process during the headspace sampling/analysis (Fruekilde *et al*., 1998).

### Electrophysiology

For electroantennographic analyses, the living pollinators and the floral headspace samples from an individual of *M. furusei* var. *subramosa* cultivated in the Tsukuba Botanical Garden were prepared with the same manner described above. To detect the physiologically active compounds in floral scents, we used coupled gas chromatographic-electroantennographic detection (GC-EAD) using antennae of *G. mikado* (18 males and 6 females) and the short-tongued fungus gnat *Coelosis fuscicauda* Okada (6 males and 7 females). Electrophysiological analyses of the floral headspace samples were conducted on a 7890A gas chromatograph (Agilent technologies, Santa Clara, CA, USA) equipped with an flame ionization detector (FID) and EAD system (Syntech, Hilversum, the Netherlands). The tip of an antenna was cut off to improve electric current, and its antenna was positioned across a forked probe (Syntech) using conductive gel (Spectra360 Electrode gel; Parker, Fairfield, NJ, USA). Humidified air was supplied to the antennal preparation. The GC effluent to the antenna passed through a heated transfer line set at 260 °C.

The GC was equipped with a DB-5 column (30 m × 0.25 mm × 0.25 μm film thickness; J&W Scientific, Folsom, CA, USA) heated from 40 to 280 °C at 10 °C min^−1^ and 30 °C min^−1^ to 310 °C where it was held for 5 min. Helium was used as the carrier gas at a velocity of 37.4 cm s^−1^, and the injector temperature was 250 °C. The column effluents were split (1 : 1) between the FID and the antennal preparation. The output signal from the antenna was amplified 10 × using an EAG probe (Syntech). The FID and EAD signals were simultaneously recorded and analysed with GC-EAD 2000 software (Syntech).

### Behavioural bioassay

Other than observations of their frequent adult emergence during the flowering period of *Asimitellaria* species, the life histories of pollinator fungus gnats are largely unknown. Consequently, we collected adult fungus gnats by netting (sweeping) when they were visiting or flying between *Asimitellaria* plants at site 3 or from another population on Mt. Daimonji in the city of Kyoto. All insects collected were kept individually under dark, cool (10 °C) conditions in 50-mL plastic tubes, into each of which a slip of paper saturated with a 5% sports drink (‘Pocari Sweat’ Otsuka Pharmaceutical, Tokyo, Japan) had been inserted as a food source. Two hours before the bioassay, the paper slip was removed from each plastic tube and the insect was kept under dark, warm (20–25 °C) conditions. As the volatile sources, 10 × 10 mm filter papers spiked with authentic volatile compounds or the actual floral headspace of *M. furusei* were used; air was pumped from the stem to allow a flow from the arms to the stem (flow rate: 200 mL min^−1^ arm^−1^, 400 mL min^−1^ stem^−1^). Each fungus gnat was allowed 10 min under illumination to choose between the arms of the Y-tube (stem 12 cm, arm 15 cm, inner diameter 25 mm). When the insect moved into the decision area (Fig. S1) of an arm within 10 min and stayed there for at least 30 s, we scored that arm as having been selected. When the insect made no choice between arms within a given time, we removed it and ignored the test. During the assay, we also determined whether the fungus gnats reacted to the volatiles with typical nectaring behaviour. This behaviour was readily observable: the insect engaged in continual (> 10 s) nectar seeking on the assay tube wall immediately after initiation of the volatile flow.

We used authentic compounds of ocimene and lilac aldehydes for our bioassay. However, because pure stereoisomeric standards are not commercially available, we used mixtures of the stereoisomers, lilac aldehydes (artificially synthesized by Soda Aromatic Co., Tokyo, Japan) and ocimene (Fluka, catalogue #74730; Sigma-Aldrich, ST. Louis, MO, USA). Triethyl citrate was used as solvent for dilution and for the blank sample. We generally applied 800 μg of each authentic compound to filter paper because lower concentrations greatly reduced the efficiency of the choice experiment (i.e. fungus gnats were not sufficiently activated). Nevertheless, we did repeat the assay using the actual floral headspace of *M. furusei* and a lower concentration (1 μg applied to the filter paper) of lilac aldehydes to determine whether the choice patterns made by the insects would be repeated at lower concentrations of the volatiles. The two arms of the Y-tube were alternated in each of five test runs, and the Y-tube was cleaned with ethanol and dried at 60 °C for more than 1 h between experiments. Fungus gnats were then preserved in 99.8% ethanol for later species identification.

**Fig 2:**
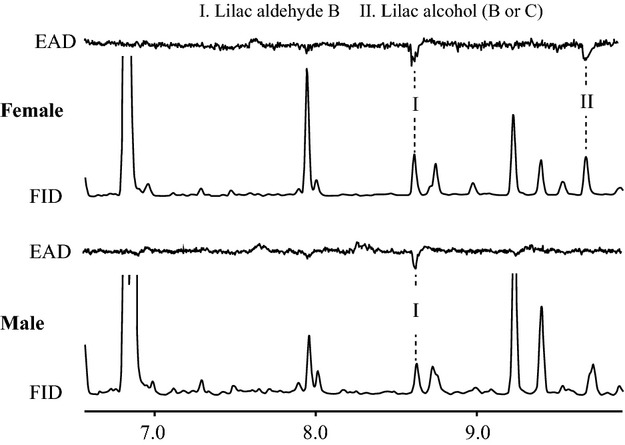
Simultaneous responses of the flame ionization detector (FID) and electroantennographic detection (EAD) using antennae of female and male *Gnoriste mikado* to scent samples of *Mitella furusei* var. *subramosa*.

Our calculations showed that applications of 800 and 1 μg of authentic compounds in our Y-tube bioassays produced concentrations that were close to 20-fold and half (designated as low. conc. in Fig.2) the atmospheric concentrations in the actual headspace of an *M. furusei* inflorescence, respectively. This calculation was based on measurements of volatile concentrations (by GC) in air collected from the stem of a Y-tube using the method for floral scent collection described above.

### Phylogenetic analyses

We constructed a high-resolution phylogenetic tree of *Asimitellaria* using the nucleotide sequences of nuclear ribosomal RNA gene spacers (ETS and ITS), and seven putative nuclear protein-coding gene orthologs (*GSII-A1*, *GSII-A2*, *PCK-A1*, *PCK-A2*, *GBSSI-A1*, *GBSSI-A2* and *GBSSI-B2*) and one pseudogene orthologue (*GBSSI-B1*) from 13 species of *Asimitellaria* that had been sequenced in a previous study (Okuyama *et al*., 2012). We determined the root of the tree using *Mitella pentandra* and *Tellima grandiflora* as outgroups because they are the closest diploid relatives of *Asimitellaria*, which is an allotetraploid lineage. Because these outgroup taxa are diploid and therefore have half a copy number each of *GSII*, *PCK-A*, *GBSSI-A*, and *GBSSI-B*, we only used the nucleotide sequences of ETS and ITS for these species. The outgroup species were removed in subsequent analyses. The total length of the aligned nucleotide sequence was 8082 bp, of which 511 bp was variable. A maximum likelihood (ML) tree search under the optimal base substitution model selected by modeltest 3.7 software (Posada & Crandall, 1998; Tamura–Nei model with gamma-shape parameter = 0.0220) was conducted using paup4.0b10 (Swofford, 2003) with tree–bisection–reconnection branch swapping starting from 100 replicates using the random addition sequence method with the MulTrees option in effect. Bootstrapping with 1000 replicates was also performed to obtain the branch support values using the same settings as in the original tree searches except that we reduced additional sequence replicates to 10. The branch length of the resulting unrooted tree (phylogenetic path distance) was calculated using a perl script (distontree: available at http://www.fifthdimension.jp/products/distontree/) and used to evaluate the association between phylogenetic and floral scent chemical relatedness among species. The aligned data matrix was deposited in the TreeBASE database, http://www.treebase.org (submission ID: 13844).

To verify the robustness of the topology of the phylogenetic tree, we also performed a maximum parsimony (MP)-based phylogenetic tree construction and bootstrapping, using heuristic search with the settings used in a previous study (Okuyama & Kato, 2009).

To count the minimum number of floral scent changes in the phylogeny, the resulting phylogenetic tree was subjected to the parsimony-based character state reconstruction using Mesquite 2.72 (Maddison & Maddison, 2009). Each of the *Asimitellaria* species was categorized into two based on the presence or absence of lilac aldehydes in their floral scents. Presence and absence were defined in the following two ways. First, species containing lilac aldehydes comprising more than 10% of the total floral scent compositions were only regarded as lilac aldehydes present, and the others, all of which contain lilac aldehydes comprising 0–4% of the total floral scent compositions, were regarded as lilac aldehydes absent (relaxed coding). Alternatively, those containing any (nonzero) lilac aldehydes were all regarded as lilac aldehydes present (strict coding).

### Analysis of correlations among traits

The dissimilarities in floral scent characters and pollinator assemblages among *Asimitellaria* species were calculated using the Bray–Curtis distance based on the relative GC peak areas of the floral compounds in each sample (Bray & Curtis, 1957) using the ‘vegdist’ procedure in the vegan package of R version 2.13.0 software (Oksanen *et al*., 2009; R Core Team, 2013). Subsequently, the correlations between floral scent characters, pollinator assemblages and phylogenetic distance were examined using a partial matrix correspondence test (PMCT) (Manly, 1986). To visualize the differences in floral scents among samples, we performed a nonmetric multidimensional scaling (NMDS) ordination analysis using the Bray–Curtis dissimilarity index.

We searched for particular floral scent compounds that were likely to be involved in the pollinator specificity of *Asimitellaria* species by examining each compound to determine whether its variation in relative abundance was correlated with pollination characters among species. For each species, the composition proportion (%) of each compound was used as a floral scent character value, and the visitation proportion (%) of either *Gnoriste* or ST fungus gnats was used as the pollinator character. For each compound, the significance of the nonzero coefficient in a general linear regression (intercept set to zero) towards the pollinator character was examined using data corrected by phylogenetically independent contrasts (PIC) (Felsenstein, 1985) with a cut-off *P*-value of 0.05. All statistical analyses were performed under the computing environment R using the ‘ape’ and ‘gee’ packages (Paradis *et al*., 2004; Carey, 2012).

## Results

### Strict pollinator specificity of *Asimitellaria* species in the sympatric populations

A total of 148 h (16 human and 132 camera hours) of field observations confirmed a lack of overlap in pollinator fungus gnat species between sympatric species of *Asimitellaria* for three independent species pairs (Table1). No hybrids were found across the three study sites.

**Table 1:** Contrasting pollinator visits between a sympatric pair of *Asimitellaria* species

	*Gnoriste mikado*	Short-tongued gnats	Others*
Site 1 (2 h, direct observation)			
*Mitella yoshinagae* (*N*† = 16)	0	6	0
*Mitella stylosa* var. *makinoi* (*N* = 13)	39	0	0
Site 2 (6 h, direct observation)			
*Mitella yamato* (*N* = 15)	0	69	1
*Mitella furusei* var. *subramosa* (*N* = 18)	102	0	0
Site 3 (132 h, time-lapse photography)			
*Mitella pauciflora* (*N* = 1)	0	33	10
*M. furusei* var. *subramosa* (*N* = 1)	36	0	11

Empididae and Syrphidae that are contributing little to the pollination (Okuyama *et al*., 2004).

Number of plant individuals observed.

### Floral scent profiles of *Asimitellaria*

Overall, we detected 27 volatile compounds in headspace samples of 13 *Asimitellaria* species, 23 of which were terpenoids (see Supporting Information, Table S1). Only linalool and (*Z*)-linalool oxide (pyranoid) were common to all species. In two-dimensional NMDS ordination plot of floral scent samples, plant species pollinated specifically by *G. mikado* and those pollinated specifically by short-tongued gnats were closely grouped in coordinate space (Fig.1b); the former contained lilac aldehydes, and the latter contained linalool as the principal volatile compound (25.7–57.6% and 19.7–93.9% of total volatiles, respectively; Fig.1c).

### Electroantennographic and behavioural response of the pollinators to the floral scent compounds

Given the complexity of the floral scent, a fundamental challenge is identifying which of the many floral scent compounds are involved in pollinator attraction and which potentially mediate pollinator specificity. To help overcome this obstacle, we conducted GC-EAD analysis and found that, among the scent compounds contained in the floral headspace of *M. furusei* var. *subramosa*, only one compound, lilac aldehyde B, was always found to elicit responses in the olfactory receptors of both male (*n* = 18) and female (*n* = 6) *G. mikado* (Fig.2). Lilac alcohol (B or C) elicited antennal responses in only female *G. mikado* in several GC-EAD runs (*n* = 6; Fig.2). In contrast, no visible responses to volatile scent compounds were detected in any GC-EAD runs using the short-tongued gnats (*C. fuscicauda n* = 3, see Supporting Information, Fig. S2).

**Fig 3:**
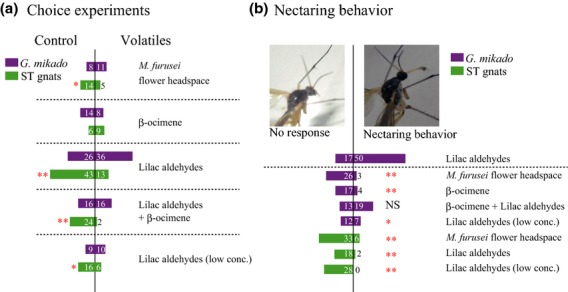
Contrasting reactions observed in the pollinators. (a) Lilac aldehydes and floral headspace of *Mitella furusei* were avoided only by short-tongued (ST) gnats (the numbers of choices are shown adjacent to the bars). One-tailed binomial test, *P < 0.05, **P < 0.001. (b) Only lilac aldehydes presented to *Gnoriste mikado* at high concentration induced typical nectaring behaviour (above the dashed line) more frequently than others (the numbers of responses are shown adjacent to the bars). Fisher's exact test, *P < 0.05, **P < 0.001.

Y-tube bioassays further revealed that lilac aldehydes were avoided by short-tongued gnats but not by *G. mikado* (Fig.3a), whereas lilac aldehydes triggered the characteristic nectaring behaviour of *G. mikado* much more frequently than other scent compounds (Fig.3b). Moreover, lilac aldehydes did not trigger nectaring behaviour in short-tongued gnats as frequently as they did in *G. mikado* (Fig.3b). The actual flower headspace of *M. furusei* and lilac aldehydes at low concentrations were also weakly avoided by short-tongued gnats (Fig.3a), although neither elicited nectaring behaviour of *G. mikado* (i.e. significantly less frequent, compared to the lilac aldehydes at higher concentration; Fig.3b).

### Phylogeny and the parallel chemical switches of *Asimitellaria*

**Fig 4:**
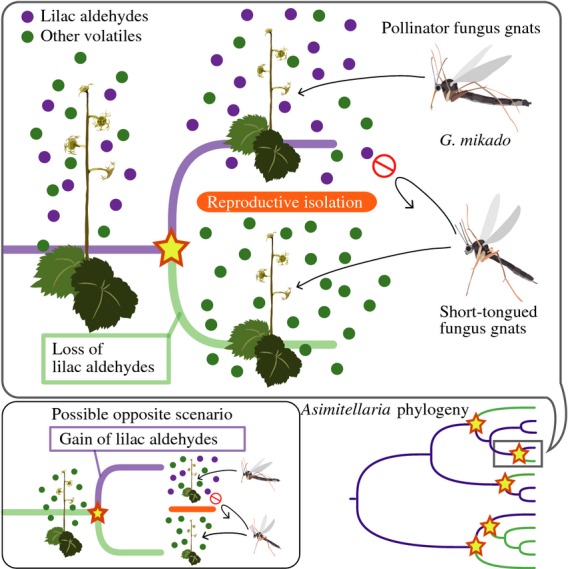
Model of recurrent speciation in *Asimitellaria* through loss/gain of lilac aldehydes. A loss or gain of lilac aldehydes can lead to reproductive isolation (and consequently speciation) through the attraction of different pollinators. The stars shown in the phylogeny at bottom right indicate where the floral scent changes may have occurred, under the assumption that the presence of lilac aldehydes is ancestral and that the loss of them is derived (based on ‘relaxed coding’). A possible opposite scenario is also shown at bottom left. Based on maximum parsimony ancestral state reconstruction, at least five scent changes are necessary to explain the phylogenetic relationships of *Asimitellaria* species with or without lilac aldehydes (Fig.1c).

The present phylogenetic analysis based on 8082-bp aligned nucleotides resulted in a fully resolved ML tree (–logL = 15,462.18), in which 9 of 12 nodes were supported with > 80% bootstrap values (Fig.1c). The MP tree search recovered the tree topology identical to the ML tree, and 10 (including all those supported in the ML tree) of 12 nodes were supported with > 80% bootstrap values. Most notably, the species that contained lilac aldehydes in their floral scents were scattered across the phylogeny, indicating that floral scent changes occurred multiple times in *Asimitellaria* speciation. Specifically, the parsimony-based character state reconstruction revealed that at least five (41.6% of the internal nodes; ‘relaxed coding’) or three (25% of the internal nodes; strict coding) scent changes coincided with the lineage splits in the phylogeny (Fig.4). The results were robust even when phylogenetic inconsistency was taken into account because the nodes with low bootstrap values only involve species with the same floral scent categories.

By referring to the phylogeny of *Asimitellaria*, we confirmed that differences in total floral scent composition among species were associated with differences in pollinator assemblages (PMCT, coefficient = 0.24, *P* < 0.001) but not with genetic distance calculated from the phylogeny (PMCT, coefficient = 0.12, *P* = 0.66). Phylogenetically independent contrasts further identified positive, significant correlations of seven volatile compounds with the rate of visitation by *G. mikado* (*F* test, *F*_1,11_ > 8.25, *P* < 0.05); among these compounds, correlations with lilac aldehyde A, B and C and 2,6-dimethyl-1,7-octadiene-3-ol still remained significant after Bonferroni correction (see Supporting Information, Fig. S3). We also identified two volatile compounds (linalool and *β*-caryophyllene) that were positively correlated with the visitation rates of short-tongued gnats (*F* test, *F*_1,11_ > 7.02, *P* < 0.05). These correlations, however, did not remain significant after Bonferroni correction.

## Discussion

### Pollinator isolation between sympatric *Asimitellaria* species

Our study revealed that the *Asimitellaria* species occurring in sympatry were almost completely differentiated in the association with pollinating fungus gnats (Table1). In addition to the pollinator visits recorded in Table1, note that at site 3, where a closely related species pair (*M. pauciflora* and *M. furusei* var. *subramosa*) exists, we collected > 100 pollinator individuals for bioassay and GC-EAD across the 6 years of the study (2009–2014). We have never observed pollinators visiting the ‘wrong’ species at that collection site.

Note that we could find no hybrids at the study sites 1–3. Because *Asimitellaria* species can easily produce hybrids if crossed artificially (Okuyama & Kato, 2009), our observation suggests that there is a strong, premating reproductive isolation between the pair of these sympatric *Asimitellaria* species. Although prepollination isolation mechanisms include other factors such as spatial isolation (geographic or habitat) and temporal (flowering time) isolation (Whitehead & Peakall, 2014), these mechanisms are obviously incomplete between the *Asimitellaria* species. Therefore, one can reasonably assume that the pollinator specificity is the major isolation mechanism and that it had a strong impact on *Asimitellaria* speciation, although a careful quantification of the individual potential reproductive barriers is needed to conclusively demonstrate this idea (Ramsey *et al*., 2003; Whitehead & Peakall, 2014).

### Chemical basis of the recurrent pollinator switchings in *Asimitellaria*

In the present study, we identified that lilac aldehydes are the scent compounds that most likely determine the pollinator specificity in *Asimitellaria* by stimulating the nectaring behaviour of *G. mikado* while repulsing short-tongued gnats. Meanwhile, linalool and/or *β*-caryophyllene might be involved in attracting the short-tongued gnats as their composition had a positive correlation with the visitation by these pollinators, although we could not confirm this through GC-EAD analysis or behavioural bioassay.

The more striking finding is that divergence of the pollination system paralleled differences in the composition of the floral scent, especially the lilac aldehydes (Fig.1). The monoterpenoids lilac aldehydes are a mixture of stereoisomers produced simultaneously through the same biosynthetic pathways, starting from *S-*(+)-linalool in *Syringa vulgaris* (lilac) (Burkhardt & Mosandl, 2003; Kreck *et al*., 2003), suggesting that production is also controlled by the same set of genes in *Asimitellaria*. Therefore, the observed variation in the pollinator assemblages of *Asimitellaria* is most likely generated by the loss or gain of the ability to synthesize lilac aldehydes, as these compounds have a dual function, namely the activation of nectaring behaviour of one pollinator and the repulsion of the other. The loss or gain of the ability to emit lilac aldehydes in the ancestral lineage of either of the species would have allowed them to maintain their genetic differentiation even under sympatric occurrence, and thus, it likely triggered or completed their speciation (Fig.4). Note, however, our behavioural experiments, based primarily on the higher dosage of the compounds, only partly supported this idea. Thus, the present hypothesis should be confirmed further in modified experimental designs, such as baiting trials in the field using artificial flowers and/or synthetic versions of the floral scents (Peakall *et al*., 2010; Whitehead & Peakall, 2014).

Although pollinators are well recognized as the major driver for the diversification of floral phenotypes, their roles as isolating barriers in sympatric species are not well established (Johnson, 2010; Van der Niet *et al*., 2014). In most systems reported so far, some extents of pollinator sharing in recently diverged plant lineages are rules rather than exceptions (Ramsey *et al*., 2003). However, in this study, we revealed that at least some pairs of closely related *Asimitellaria* species were pollinated by nonoverlapping pollinator species and that the specific set of floral scent compounds, lilac aldehydes, may be responsible for the pollinator differences. A similar finding has been reported from several sexually deceptive, terrestrial orchid systems including European *Ophrys* and Australian *Chiloglottis*, in which a subtle difference in floral scent chemicals underlies the strong pollinator isolation (Stökl *et al*., 2009; Peakall *et al*., 2010; Xu *et al*., 2011; Whitehead & Peakall, 2014). Likewise, floral scents are likely to determine the specificity of *Glochidion–Epicephala* obligate pollination mutualisms in sympatric populations (Okamoto *et al*., 2007), and in French Guiana, three sympatric, morphologically unspecialized aroid species each attract a different set of euglossine bees by a differentiated composition of floral scents (Hentrich *et al*., 2010). Taken together, speciation through pollinator shifts might be more widespread in specialized plant–pollinator systems mediated by floral scents. Pollinator species could dramatically change through the simple switching of floral scent compounds by the plants (Shuttleworth & Johnson, 2010). Because empirical studies regarding the process of pollinator-driven speciation are still scarce (Van der Niet & Johnson, 2012), the *Asimitellaria*–fungus gnat system would serve as an excellent model to address this issue, especially using the tools and methodologies of genetics (Okuyama & Akashi, 2013).

Determining the evolutionary paths among apparently discontinuous reproductive traits exhibited by closely related species has been a major challenge in evolutionary biology (Coyne & Orr, 2004; Whibley *et al*., 2006). In floral biology, different pollinators are presumed to be associated with different suites of floral characters. Thus, coordinated changes in multiple floral traits have been considered necessary for the pollinator shifts (Dell'Olivo & Kuhlemeier, 2013). In contrast, in this study, we hypothesized that lilac aldehydes might enable an instantaneous change of pollinators, presumably without passing through unfit intermediates, because the compounds have contrasting effects on the different pollinators. In a more general context, pleiotropic functions of a single secondary metabolite such as in lilac aldehydes may be more widespread and could partly account for the diversification of organisms through saltational adaptation to various environments with different assemblages of interacting organisms.

## Supporting information

**Figure S1** An illustration of a Y-tube assay system.

**Figure S2** Simultaneous responses of the flame ionization detector (FID) and electroantennographic detection (EAD) using antennae of female and male short-tongued fungus gnats (*Coelosia fuscicauda*) to scent samples of *M. furusei* var. *subramosa*.

**Figure S3** A phylogenetically corrected association plot between the relative amount of the 27 constituent compounds of *Asimitellaria* floral scent and the rate of visitation by *G. mikado* (unit: %). Assuming per cent values as the substitutes for the frequency of pollinator visits and/or the amount of scent emission, they can exceed 100 because the comparisons were made with the branch length standardized.

**Table S1** Floral scent compounds of the 13 *Asimitellaria* species expressed as relative and absolute amounts (Average ± SD).
