# Supplementary material for: Parallel chemical switches underlying pollinator isolation in Asian Mitella
Source: J Evol Biol. 2015 Feb 20;28(3):590–600. doi: 10.1111/jeb.12591 (PMC4418413; doi:10.1111/jeb.12591)
Supplement: Supplementary file 3 — Figure S3 A phylogenetically corrected association plot between the relative amount of the 27 constituent compounds of Asimitellaria floral scent and the rate of visitation by G. mikado (unit: %). Assuming per cent values as the substitutes for the frequency of pollinator visits and/or the amount of scent emission, they can exceed 100 because the comparisons were made with the branch length standardized. [file jeb0028-0590-sd3.pdf]

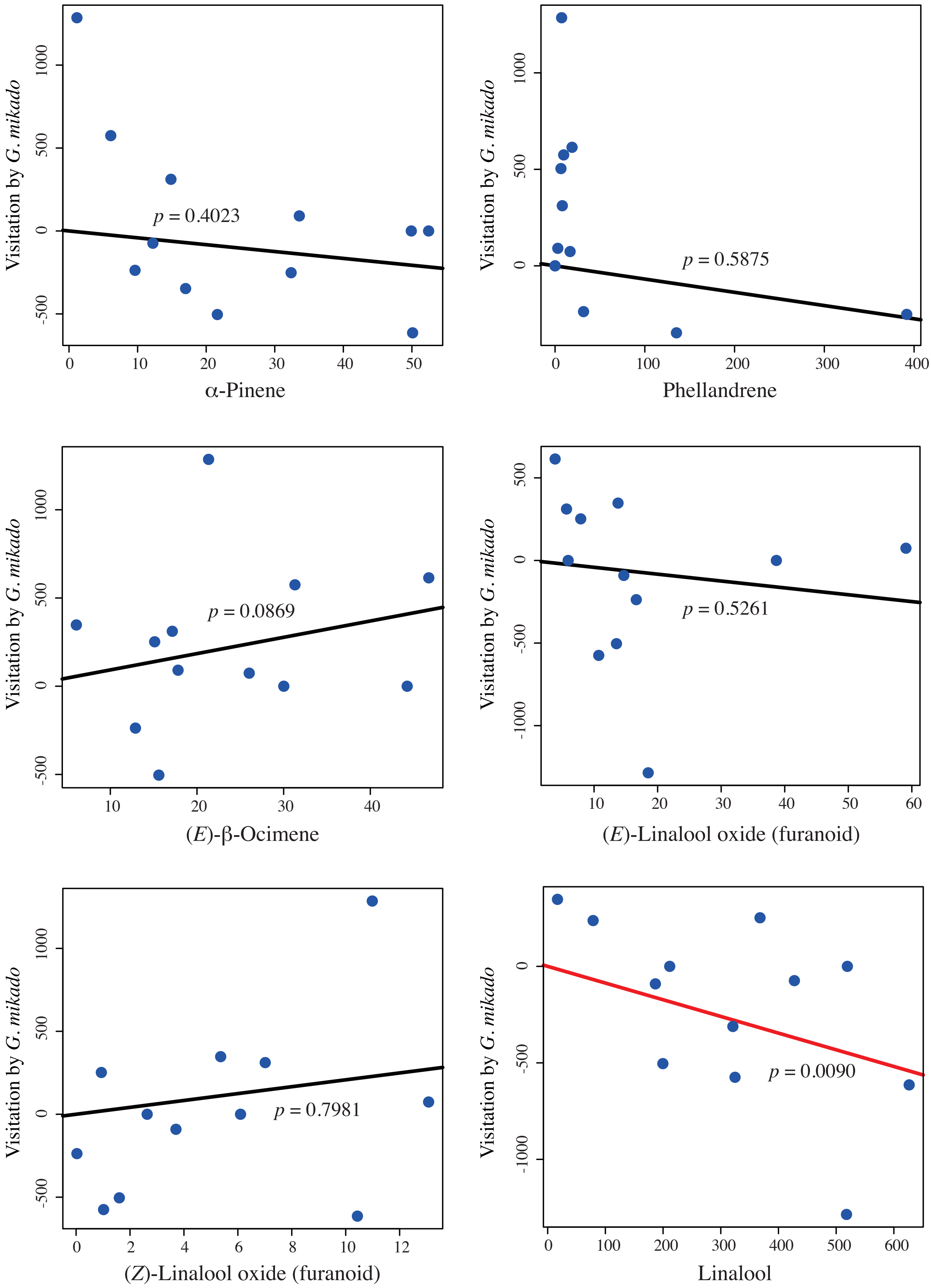

Figure S3. A phylogenetically corrected association plot between the relative amount of the 27 constituent compounds of *Asimitellaria* floral scent and the rate of visitation by *G. mikado* (unit: %). Assuming percent values as the substitutes for the frequency of pollinator visits and/or the amount of scent emission, they can exceed 100 because the comparisons were made with the branch length standardized.

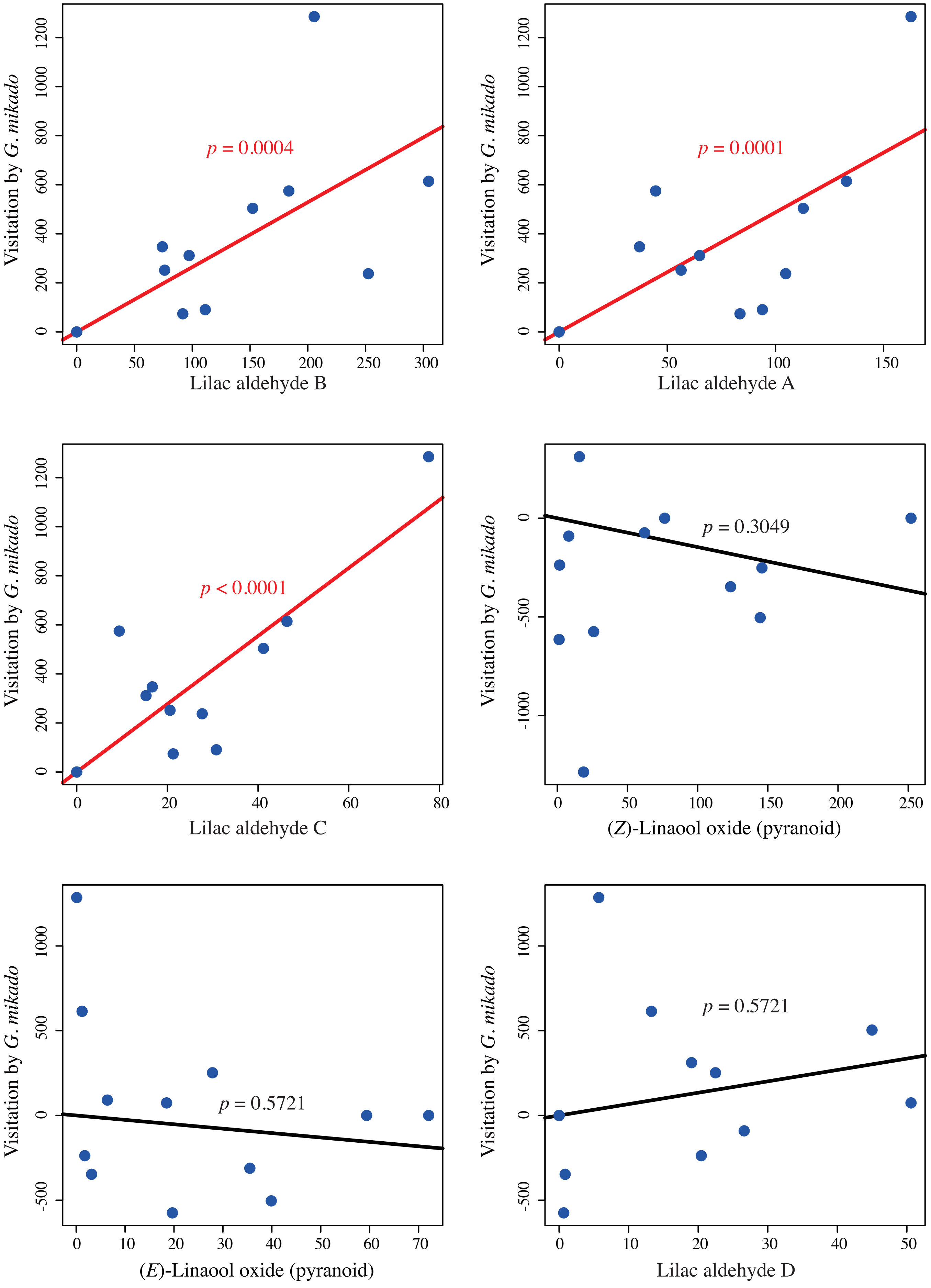

Figure S3. Continued.

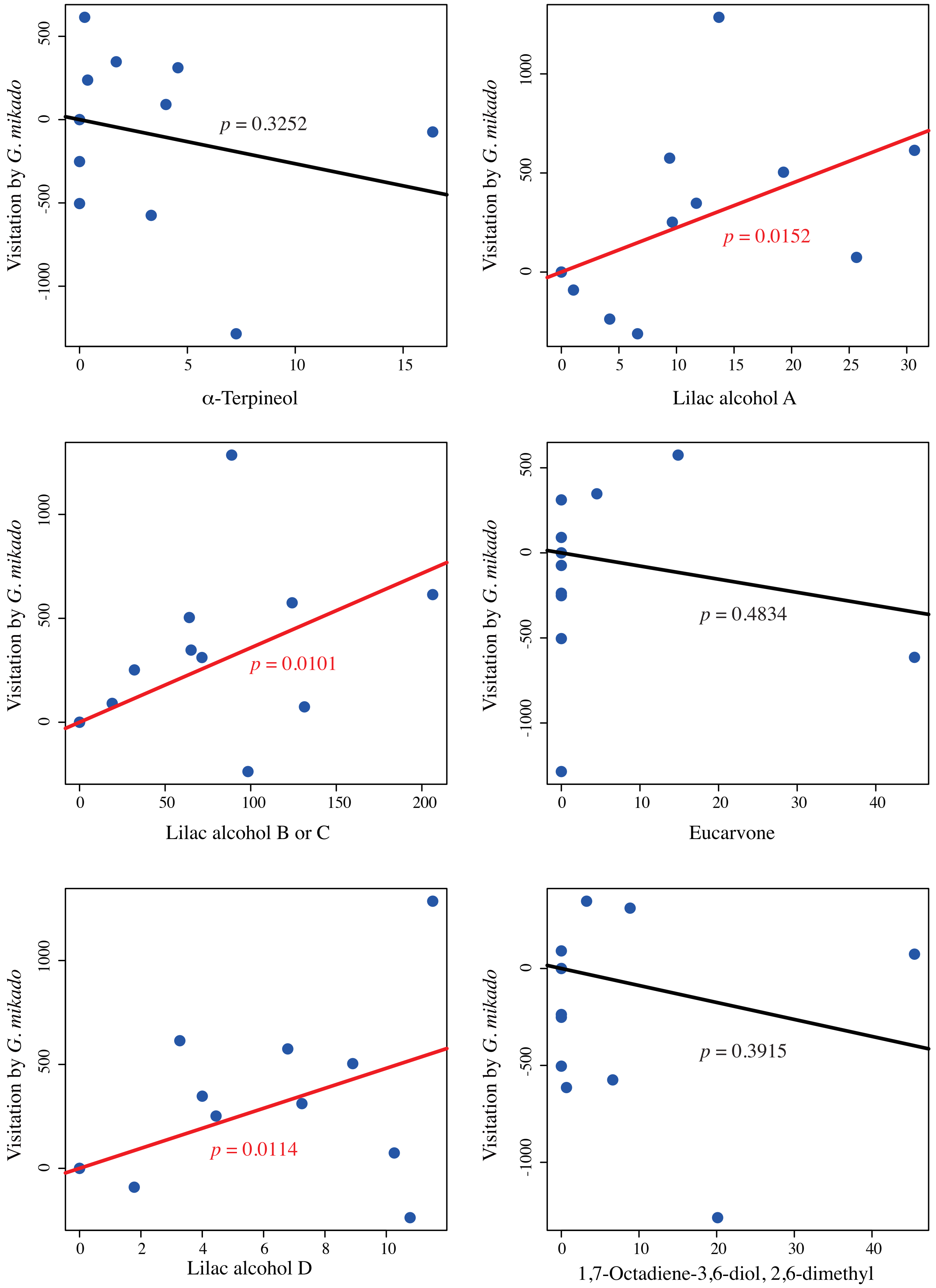

Figure S3. Continued.

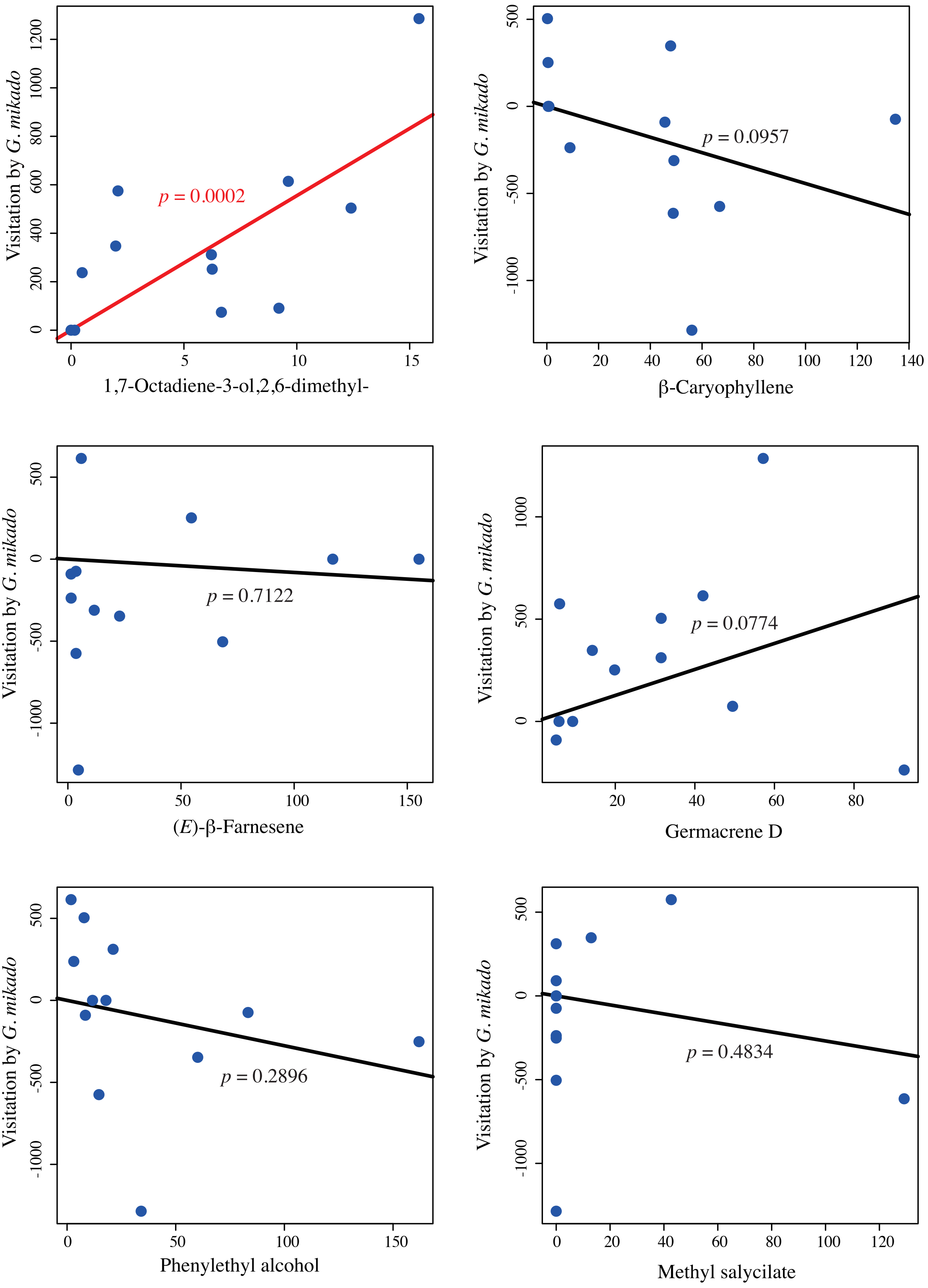

Figure S3. Continued.

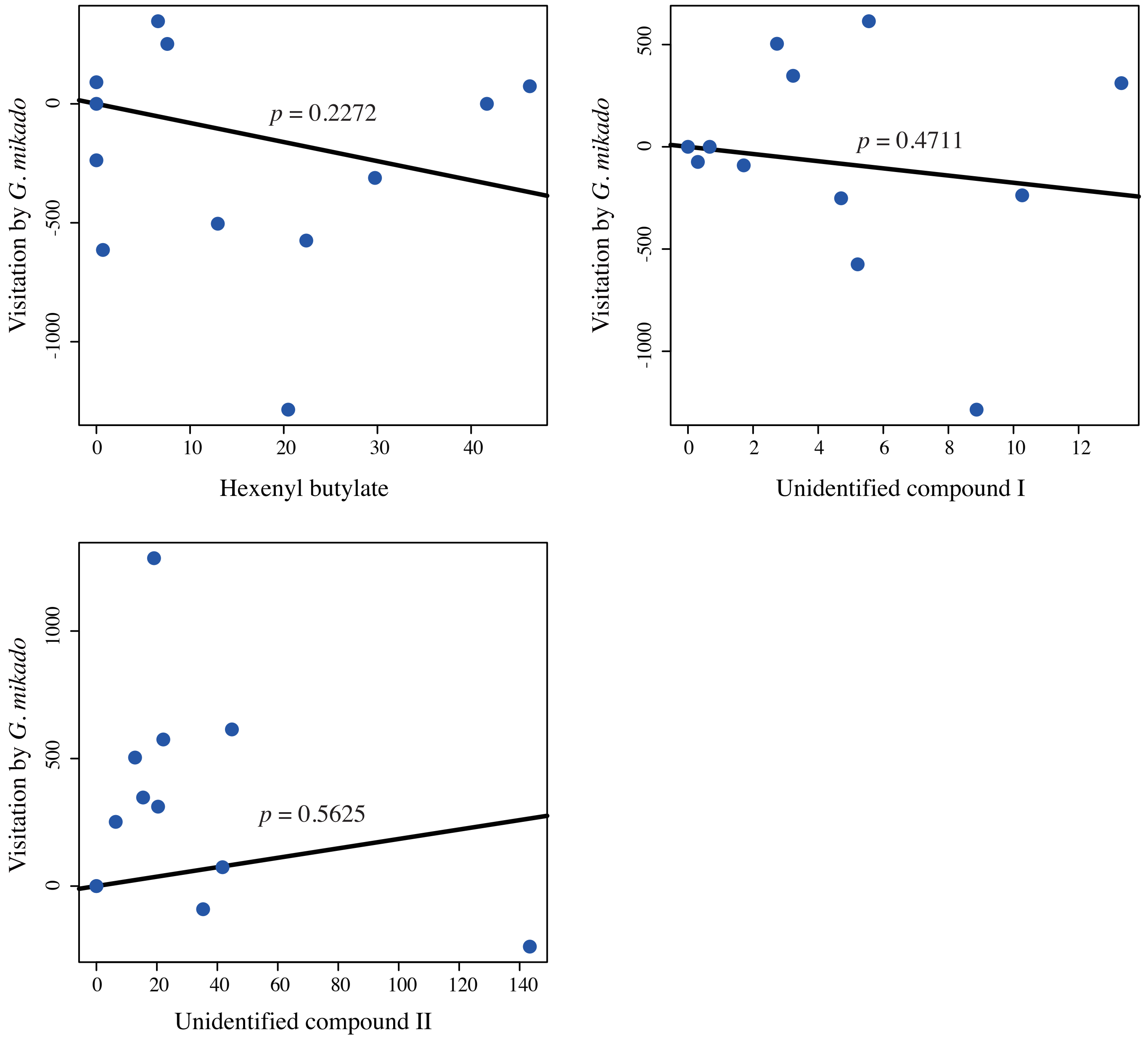

Figure S3. Continued.
