# Supplementary material for: Parallel chemical switches underlying pollinator isolation in Asian Mitella
Source: J Evol Biol. 2015 Feb 20;28(3):590–600. doi: 10.1111/jeb.12591 (PMC4418413; doi:10.1111/jeb.12591)
Supplement: Supplementary file 4 — Table S1 Floral scent compounds of the 13 Asimitellaria species expressed as relative and absolute amounts (Average ± SD). [file jeb0028-0590-sd4.pdf]

Table S1. Floral scent compounds of the 13 *Asimitellaria* species expressed as relative and absolute amounts (Average ± SD).

Compound	RI[a]	*M. stylosa* var. *stylosa* *N*=9		*M. stylosa* var. *makinoi* *N*=7		*M. furusei* var. *furusei* *N*=3		*M. furusei* var. *subramosa* *N*=20	
		Rerative amount	Absolute amount[e]	Rerative amount	Absolute amount[e]	Rerative amount	Absolute amount[e]	Rerative amount	Absolute amount[e]
Terpenoid									
α-Pinene[b]	931	0.17±0.24	0.4±0.6	0.73±1.20	0.1±0.2	0.67±1.15	0.1±0.2	3.75±8.99	0.3±0.6
Phellandrene[b]	1005	0.83±1.17	3.5±5.4	0.95±1.07	0.3±0.4	2.42±3.26	2.0±3.2	1.19±1.41	0.8±1.2
(*E*)-β-ocimene[b]	1056	4.37±5.66	20.0±31.0	1.91±2.14	0.4±0.6	3.81±3.27	2.5±3.3	3.45±4.49	2.2±3.1
(*E*)-Linalool oxide (furanoid)[b]	1085	0.95±1.16	4.4±7.1	2.41±2.11	0.3±0.2	0.67±0.71	0.6±0.7	1.97±2.65	1.3±1.8
(*Z*)-Linalool oxide (furanoid)[b]	1104	0.28±0.42	0.5±0.6	1.43±1.31	0.2±0.2	0.20±0.32	0.2±0.3	1.03±2.11	0.4±1.4
Linalool[b]	1101	11.96±11.94	26.7±19.1	26.20±10.18	4.8±4.3	5.49±2.24	4.4±4.4	8.36±8.82	6.8±10.9
Lilac aldehyde B[b]	1142	35.89±12.79	152.0±149.8	14.05±8.67	2.3±2.1	13.97±7.55	10.2±9.7	24.34±12.74	43.0±81.3
Lilac aldehyde A[b]	1150	15.57±2.91	62.5±56.0	11.28±9.88	1.7±2.3	10.34±7.63	10.4±14.3	19.58±8.65	29.9±55.0
Lilac aldehyde C[b]	1165	5.31±1.10	21.5±19.0	5.24±3.32	0.8±0.8	4.05±2.03	4.2±4.7	7.87±4.21	12.7±27.2
(*Z*)-linaool oxide (pyranoid)[b]	1175	0.09±0.05	0.3±0.3	2.22±2.74	0.3±0.2	0.21±0.06	0.2±0.1	1.36±2.18	1.2±3.0
(*E*)-linalool oxide(pyranoid)[b]	1179	0.24±0.25	1.1±1.5	0.48±0.65	0.1±0.1	0.28±0.43	0.1±0.1	1.04±2.58	0.5±1.1
Lilac aldehyde D[c]	1181	0.86±1.87	2.8±5.2	2.85±4.19	0.7±1.2	1.82±2.78	1.6±2.6	0.29±0.55	0.1±0.2
α-Terpineol[b]	1195	0.03±0.02	0.1±0.1	–	–	–	–	0.36±1.32	0.2±0.4
Lilac alcohol A[c]	1203	2.86±1.18	8.7±6.5	1.82±1.65	0.3±0.3	3.43±0.81	3.2±2.6	1.63±1.50	1.9±3.0
Lilac alcoholB or C[c]	1215	16.94±6.78	56.3±39.5	9.90±8.45	1.8±1.7	27.94±7.20	23.8±22.1	11.27±10.35	15.5±23.4
Eucarvone[c]	1223	–	–	–	–	–	–	–	–
Lilac alcohol D[c]	1240	0.75±0.20	3.1±3.1	1.20±0.69	0.2±0.4	1.42±1.04	1.3±1.1	0.99±0.82	1.3±2.5
Unidentified monoterpene [m/z 68(100), 43(58), 85(48), 55(36), 96(17), 152(0.6)][d]	1270	–	–	–	–	–	–	–	–
2,6-dimethyl-1,7-Octadiene-3,6-diol[c]	1276	0.91±0.65	4.9±7.1	1.02±0.75	0.1±0.1	1.22±0.75	1.4±1.4	1.82±3.27	1.5±2.2
β-Caryophyllene[b]	1426	0.21±0.53	0.2±0.3	4.61±4.47	1.0±1.0	1.16±0.85	0.9±1.1	0.84±2.70	0.3±0.5
(*E*)-β-farnesene[b]	1456	–	–	0.24±0.30	0.1±0.1	1.27±1.35	0.5±0.5	0.12±0.28	0.1±0.1
Germacrene D[c]	1487	1.24±1.12	5.3±9.5	5.88±8.16	1.9±4.3	10.37±2.65	10.0±8.5	6.68±9.33	6.7±13.0
Benzenoid									
Phenylethyl Alcohol[b]	1113	0.27±0.24	1.3±2.0	0.84±1.17	0.1±0.1	–	–	0.03±0.13	0.1±0.3
Methyl salicylate[b]	1191	–	–	–	–	–	–	–	–
Aliphatics									
3-Hexenyl butyrate[c]	1190	–	–	–	–	–	–	–	–
Unidentified Compounds									
unidentified compound I [m/z 111(100), 43(52), 67(48), 55(48), 71(35), 99(24), 83(19), 126(13)][d]	1053	0.25±0.21	0.7±0.9	0.93±0.87	0.1±0.1	0.89±0.45	0.6±0.4	0.86±1.38	0.3±0.4
unidentified compound II [m/z 98(100), 57(75), 43(60), 84(34), 140(18), 113(15), 71(14)][d]	1055	–	–	3.80±6.15	1.3±2.4	8.39±12.83	7.2±12.1	1.15±3.53	0.3±0.9

[a] Retention indices relative to n-alkanes on the Rtx-5Sil column.

[b] Idetified baced on mass spectrum and retention index of authentic compound.

[c] Identified by similarity of mass spectrum to those in the libraries and previously reported RI index in the NIST Chemistry Web

[d] Typical fragment ions and their intensities are given for unidentified compounds.

[e] Mean amount (ng) of floral volatile per three hours per individual.

Table S1. Continued.

Compound	RI[a]	*M. japonica* *N*=18		*M. koshiensis* *N*=8		*M. kiushiana* *N*=8		*M. acerina* *N*=9	
		Rerative amount	Absolute amount[d]	Rerative amount	Absolute amount[e]	Rerative amount	Absolute amount[e]	Rerative amount	Absolute amount[e]
Terpenoid									
α-Pinene[b]	931	0.87±1.67	0.3±0.7	1.28±2.06	0.1±0.2	5.07±13.20	0.03±0.03	0.37±1.10	0.01±0.02
Phellandrene[b]	1005	0.65±1.78	0.6±2.0	1.13±1.43	1.9±3.6	–	–	–	–
(*E*)-β-ocimene[b]	1056	1.12±1.83	0.8±2.1	1.88±2.56	3.0±5.2	0.43±1.09	0.01±0.01	–	–
(*E*)-Linalool oxide (furanoid)[b]	1085	–	–	5.23±3.55	5.3±11.2	1.08±0.36	1.4±2.3	1.84±2.79	0.1±0.1
(*Z*)-Linalool oxide (furanoid)[b]	1104	–	–	0.99±0.76	1.4±2.8	1.24±0.72	1.7±2.7	0.21±0.31	0.1±0.1
Linalool[b]	1101	42.72±20.76	26.2±47.9	41.89±21.78	30.0±51.5	67.55±16.06	75.0±110.3	71.38±16.60	18.6±29.6
Lilac aldehyde B[b]	1142	15.11±13.16	5.3±6.5	6.21±7.77	3.6±4.1	0.41±0.39	0.3±0.5	0.10±0.28	0.01±0.01
Lilac aldehyde A[b]	1150	11.20±6.06	3.3±3.1	5.65±4.81	2.5±2.2	0.26±0.38	0.2±0.3	1.55±2.31	0.5±1.2
Lilac aldehyde C[b]	1165	4.09±2.79	1.2±1.2	1.44±1.46	0.8±1.0	0.22±0.36	0.04±0.04	1.61±1.89	0.04±0.05
(*Z*)-linaool oxide (pyranoid)[b]	1175	1.02±1.33	0.4±0.5	1.06±0.92	1.4±3.2	0.25±0.51	0.2±0.3	1.26±2.60	0.1±0.3
(*E*)-linalool oxide(pyranoid)[b]	1179	0.42±1.20	0.1±0.1	1.34±1.07	1.6±3.5	0.18±0.20	0.5±0.8	3.99±9.24	0.1±0.2
Lilac aldehyde D[c]	1181	4.46±13.60	1.5±4.1	3.42±4.43	3.4±6.3	0.23±0.44	0.4±1.1	–	–
α-Terpineol[b]	1195	–	–	0.06±0.08	0.2±0.4	–	–	–	–
Lilac alcohol A[c]	1203	1.91±1.87	0.6±1.1	1.73±1.68	1.7±2.5	0.15±0.18	0.2±0.4	1.84±2.72	0.1±0.2
Lilac alcoholB or C[c]	1215	6.36±4.52	3.1±4.3	8.88±9.10	8.5±12.3	0.74±1.05	0.7±1.5	0.20±0.59	0.03±0.07
Eucarvone[c]	1223	–	–	–	–	4.15±1.79	3.1±5.4	–	–
Lilac alcohol D[c]	1240	0.88±1.01	0.7±1.5	0.69±0.58	1.0±1.9	0.76±2.12	3.5±9.9	–	–
Unidentified monoterpene [m/z 68(100), 43(58), 85(48), 55(36), 96(17), 152(0.6)][d]	1270	–	–	3.07±4.44	7.3±19.6	0.06±0.13	0.04±0.09	–	–
2,6-dimethyl-1,7-Octadiene-3,6-diol[c]	1276	1.24±1.19	0.5±0.6	0.45±0.35	0.5±0.8	0.01±0.02	0.01±0.02	0.10±0.30	0.002±0.006
β-Caryophyllene[b]	1426	0.05±0.13	0.04±0.1	2.32±3.55	0.6±1.2	4.98±3.00	9.2±18.4	10.03±8.42	0.8±0.8
(*E*)-β-farnesene[b]	1456	1.34±2.38	0.6±1.2	0.39±0.27	0.3±0.6	0.08±0.11	0.2±0.5	1.47±1.68	0.6±1.5
Germacrene D[c]	1487	3.50±4.59	3.9±12.5	3.41±4.13	7.6±15.9	0.06±0.08	0.2±0.3	0.44±1.17	0.1±0.1
Benzenoid									
Phenylethyl Alcohol[b]	1113	1.48±2.54	0.5±0.9	0.02±0.04	0.01±0.02	0.01±0.03	0.002±0.005	–	–
Methyl salicylate[b]	1191	–	–	–	–	11.93±7.72	15.3±24.2	–	–
Aliphatics									
3-Hexenyl butyrate[c]	1190	–	–	3.13±4.28	3.4±7.4	0.06±0.18	0.2±0.5	3.61±5.98	0.8±1.4
Unidentified Compounds									
unidentified compound I [m/z 111(100), 43(52), 67(48), 55(48), 71(35), 99(24), 83(19), 126(13)][d]	1053	0.29±0.55	0.3±0.9	1.50±1.53	3.4±8.5	0.03±0.05	0.1±0.2	–	
unidentified compound II [m/z 98(100), 57(75), 43(60), 84(34), 140(18), 113(15), 71(14)][d]	1055	1.27±3.61	2.3±9.4	2.82±3.45	4.6±8.7	0.03±0.06	0.1±0.1	–	

[a] Retention indices relative to n-alkanes on the Rtx-5Sil column.

[b] Idetified baced on mass spectrum and retention index of authentic compound.

[c] Identified by similarity of mass spectrum to those in the libraries and previously reported RI index in the NIST Chemistry Web

[d] Typical fragment ions and their intensities are given for unidentified compounds.

[e] Mean amount (ng) of floral volatile per three hours per individual.

Table S1. Continued.

Compound	RI[a]	*M. pauciflora* *N*=13		*M. yoshinagae* *N*=8		*M.*"*yamato*" *N*=12		*M.*"*hayato*" *N*=9	
		Rerative amount	Absolute amount[e]	Rerative amount	Absolute amount[e]	Rerative amount	Absolute amount[e]	Rerative amount	Absolute amount[e]
Terpenoid									
α-Pinene[b]	931	2.10±7.01	0.1±0.3	–	–	7.68±19.59	0.2±0.5	2.48±7.45	0.5±1.6
Phellandrene[b]	1005	–	–	–	–	–	–	–	–
(*E*)-β-ocimene[b]	1056	0.12±0.22	0.05±0.10	–	–	2.24±3.81	1.7±3.9	5.22±8.48	0.1±0.2
(*E*)-Linalool oxide (furanoid)[b]	1085	1.06±1.70	0.2±0.3	0.98±1.18	0.5±0.6	3.84±5.39	1.3±2.9	–	–
(*Z*)-Linalool oxide (furanoid)[b]	1104	0.11±0.22	0.05±0.14	–	–	0.60±2.09	0.0004±0.001	–	–
Linalool[b]	1101	70.79±22.95	34.3±67.2	93.88±10.90	98.9±148.3	33.87±24.06	7.2±16.0	54.84±25.70	2.4±3.5
Lilac aldehyde B[b]	1142	–	–	–	–	–	–	–	–
Lilac aldehyde A[b]	1150	–	–	–	–	–	–	–	–
Lilac aldehyde C[b]	1165	–	–	–	–	–	–	–	–
(*Z*)-linaool oxide (pyranoid)[b]	1175	5.26±13.91	0.3±0.3	0.15±0.23	0.3±0.9	27.70±26.22	10.3±25.3	20.12±18.21	0.8±0.9
(*E*)-linalool oxide(pyranoid)[b]	1179	0.09±0.28	0.1±0.2	0.03±0.08	0.1±0.3	3.05±4.72	0.6±1.8	8.94±20.88	0.4±1.2
Lilac aldehyde D[c]	1181	–	–	–	–	–	–	–	–
α-Terpineol[b]	1195	1.16±1.35	0.3±0.5	–	–	–	–	–	–
Lilac alcohol A[c]	1203	–	–	–	–	–	–	–	–
Lilac alcoholB or C[c]	1215	–	–	–	–	–	–	–	–
Eucarvone[c]	1223	–	–	–	–	–	–	–	–
Lilac alcohol D[c]	1240	–	–	–	–	–	–	–	–
Unidentified monoterpene [m/z 68(100), 43(58), 85(48), 55(36), 96(17), 152(0.6)][d]	1270	–	–	–	–	–	–	–	–
2,6-dimethyl-1,7-Octadiene-3,6-diol[c]	1276	–	–	0.01±0.04	0.1±0.2	–	–	–	–
β-Caryophyllene[b]	1426	11.43±14.54	9.3±14.2	–	–	–	–	0.08±0.24	0.02±0.05
(*E*)-β-farnesene[b]	1456	0.64±1.88	0.1±0.1	1.06±2.90	0.5±1.1	21.01±19.57	14.4±44.9	5.62±6.47	0.4±0.5
Germacrene D[c]	1487	0.07±0.16	0.1±0.4	0.02±0.06	0.1±0.3	–	–	0.92±2.76	0.02±0.07
Benzenoid									
Phenylethyl Alcohol[b]	1113	5.65±13.93	2.4±6.2	–	–	–	–	1.77±4.38	0.1±0.3
Methyl salicylate[b]	1191	–	–	–	–	–	–	–	–
Aliphatics									
3-Hexenyl butyrate[c]	1190	–	–	3.80±7.22	1.6±2.6	–	–	–	–
Unidentified Compounds									
unidentified compound I [m/z 111(100), 43(52), 67(48), 55(48), 71(35), 99(24), 83(19), 126(13)][d]	1053	1.52±2.10	0.4±0.5	0.06±0.17	0.3±0.8	–	–	–	–
unidentified compound II [m/z 98(100), 57(75), 43(60), 84(34), 140(18), 113(15), 71(14)][d]	1055	–	–	–	–	–	–	–	–

[a] Retention indices relative to n-alkanes on the Rtx-5Sil column.

[b] Idetified baced on mass spectrum and retention index of authentic compound.

[c] Identified by similarity of mass spectrum to those in the libraries and previously reported RI index in the NIST Chemistry Web

[d] Typical fragment ions and their intensities are given for unidentified compounds.

[e] Mean amount (ng) of floral volatile per three hours per individual.

Table S1. Continued.

Compound	RI[a]	*M. formosana* *N*=4	
		Rerative amount	Absolute amount[e]
Terpenoid			
α-Pinene[b]	931	4.99±6.11	0.4±0.5
Phellandrene[b]	1005	36.18±8.47	1.9±1.1
(*E*)-β-ocimene[b]	1056	0.57±0.70	0.04±0.06
(*E*)-Linalool oxide (furanoid)[b]	1085	–	–
(*Z*)-Linalool oxide (furanoid)[b]	1104	–	–
Linalool[b]	1101	19.70±16.24	1.0±0.6
Lilac aldehyde B[b]	1142	–	–
Lilac aldehyde A[b]	1150	–	–
Lilac aldehyde C[b]	1165	–	–
(*Z*)-linaool oxide (pyranoid)[b]	1175	22.10±14.39	1.4±1.3
(*E*)-linalool oxide(pyranoid)[b]	1179	–	–
Lilac aldehyde D[c]	1181	–	–
α-Terpineol[b]	1195	–	–
Lilac alcohol A[c]	1203	–	–
Lilac alcoholB or C[c]	1215	–	–
Eucarvone[c]	1223	–	–
Lilac alcohol D[c]	1240	–	–
Unidentified monoterpene [m/z 68(100), 43(58), 85(48), 55(36), 96(17), 152(0.6)][d]	1270	–	–
2,6-dimethyl-1,7-Octadiene-3,6-diol[c]	1276	–	–
β-Caryophyllene[b]	1426	–	–
(*E*)-β-farnesene[b]	1456	–	–
Germacrene D[c]	1487	–	–
Benzenoid			
Phenylethyl Alcohol[b]	1113	15.89±17.07	0.5±0.3
Methyl salicylate[b]	1191	–	–
Aliphatics			
3-Hexenyl butyrate[c]	1190	–	–
Unidentified Compounds			
unidentified compound I [m/z 111(100), 43(52), 67(48), 55(48), 71(35), 99(24), 83(19), 126(13)][d]	1053	0.58±0.67	0.03±0.04
unidentified compound II [m/z 98(100), 57(75), 43(60), 84(34), 140(18), 113(15), 71(14)][d]	1055	–	–

[a] Retention indices relative to n-alkanes on the Rtx-5Sil column.

[b] Idetified baced on mass spectrum and retention index of authentic compound.

[c] Identified by similarity of mass spectrum to those in the libraries and previously reported RI index in the NIST Chemistry Web

[d] Typical fragment ions and their intensities are given for unidentified compounds.

[e] Mean amount (ng) of floral volatile per three hours per individual.
